# Risk Perception and Its Influence on Pre-Exposure Prophylaxis (PrEP) Uptake and Adherence Among Pharmacy-Based PrEP Users in South Africa: A Qualitative Study

**DOI:** 10.1177/23259582261447318

**Published:** 2026-06-09

**Authors:** Hugo Ferrero, Kelechi Elizabeth Oladimeji, Mara Aileen Yerkes, Athini Nyatela, Cheryl Hendrickson, Angela Tembo, Samanta Tresha Lalla-Edward

**Affiliations:** 1 Department of Interdisciplinary Social Science, 120705 Utrecht University, Utrecht, The Netherlands; 2 Ezintsha Research Centre, Faculty of Health Sciences, University of Witwatersrand, Johannesburg, South Africa; 3 Cluster of Excellence “Politics of Inequality”, 26567 University of Konstanz, Konstanz, Germany; 4 Centre for Social Development in Africa, 61799 University of Johannesburg, Johannesburg, South Africa; 5 Health Economics and Epidemiology Research Office (HE2RO), Faculty of Health Sciences, 590641 University of Witwatersrand, Johannesburg, South Africa; 6 Department of Global Public Health and Bioethics, Julius Global Health, Julius Center for Health Sciences and Primary Care, University Medical Center Utrecht, Utrecht University, Utrecht, The Netherlands

**Keywords:** PrEP, risk perception, HIV prevention, adherence, theory of planned behavior, protection motivation theory, South Africa, pharmacy-based PrEP programs, qualitative study

## Abstract

**Background:**

Pre-exposure prophylaxis (PrEP) is highly effective in preventing human immunodeficiency virus (HIV) transmission, particularly for individuals at increased risk. However, uptake and long-term adherence remain challenging, with limited data on pharmacies beyond pilot studies. This study explored how psychological, social, and structural factors shape perceptions of HIV risk and influence PrEP adherence, using the Tripartite Risk Perception (TRIRISK) model, Protection Motivation Theory, and the Theory of Planned Behavior.

**Methods:**

A qualitative design using in-depth interviews (IDIs) was conducted pre-implementation (May 2023) and during implementation (April-July 2024) of pharmacy-based PrEP services. Participants were adults (18+years) accessing pharmacy-based PrEP services in Gauteng and the Western Cape, South Africa. Data were analyzed thematically using Excel and MAXQDA, guided by the integrated behavioral frameworks.

**Results:**

A total of 99 IDIs were conducted, 30 in 2023, 69 in 2024. Through the TRIRISK model, this study found that decisions to start or continue PrEP were shaped by perceived vulnerability to HIV, awareness of risky sexual behaviors, mistrust of partners, and emotional experiences like fear and trauma. The PMT further highlighted how emotional triggers, along with perceived severity and coping efficacy, affected motivation to initiate or continue PrEP. The TPB helped explain how subjective norms, such as stigma and social judgment, and perceived behavioral control, shaped by access, convenience, and privacy, impacted adherence.

**Conclusion:**

Integrated behavioral frameworks offer critical insights into PrEP-related decision making. Interventions, including pharmacy-based PrEP models, should address emotional barriers, such as stigma-sensitive messaging, strengthening social support, and reducing structural inequalities.

## Plain language summary

HIV Risk Perception and PrEP Use

Pre-exposure prophylaxis (PrEP) is a highly effective medicine that helps prevent human immunodeficiency virus (HIV). However, not everyone who needs PrEP uses it effectively. In South Africa, PrEP is being offered in some pharmacies to make it easier to access compared to most public clinic settings. This study looked at what people think and feel about their risk of getting HIV, and how those thoughts shape their decision to take PrEP. We interviewed 2 groups of pharmacy clients at different stages of implementation: one group before pharmacy-based PrEP was rolled out, and another group during implementation after PrEP had been initiated. We used 3 behavioral theories to understand how people assess their risk and make decisions about PrEP use. We found that people were more likely to start PrEP when they felt personally at risk of HIV, for example due to a partner's unfaithfulness or past traumatic experiences. Support from peers, privacy at the pharmacy, and easy access made it easier to keep using PrEP. However, stigma, travel, and life challenges like family responsibilities often made it harder to continue. This study shows that providing PrEP through pharmacies can work well, but emotional, social, and structural barriers must be addressed. PrEP programs should include supportive messaging, flexible access options, and tools that help people stay on PrEP even when life gets difficult.

## Introduction

Pre-exposure prophylaxis (PrEP) is a highly effective biomedical strategy for human immunodeficiency virus (HIV) prevention that significantly reduces the risk of HIV acquisition.^1^ A pharmacokinetic study of oral PrEP estimated effectiveness to be 99% if doses are taken 7 days a week, 96% for 4 doses per week, and 76% for 2 doses per week.^2^ South Africa has one of the highest HIV prevalence rates in the world, with an estimated 13.9% of the population living with HIV in 2022.^3^ Oral PrEP is included as part of South Africa's HIV prevention package, but use remains low when compared to UNAIDS targets and early discontinuation rates are high.^4,5^ A recent systematic review identified several common barriers to PrEP uptake in Africa, including fear of side-effects, stigma, cost, low awareness, concerns about effectiveness, and unfriendly services, which contribute to limited.^6^ In response to these barriers, alternative PrEP delivery models, including pharmacy-based delivery, are increasingly being explored to improve convenience, privacy, and access.^7,8^ Pharmacy-based PrEP may reduce waiting times, decentralize services, and provide a less stigmatizing entry point for HIV prevention than some public-sector clinic settings.^7^ However, despite growing policy and programmatic interest in pharmacy-based PrEP, there remains limited qualitative evidence on how users in these settings perceive HIV risk and make decisions about PrEP uptake and continued use.^8,9^ This is important because the context in which PrEP is delivered may shape uptake and adherence differently from other service delivery models.

While these structural and interpersonal barriers are well documented, they do not fully explain the nuances of individual decision making. Emerging research highlights risk perception, how individuals assess their likelihood of HIV infection, as another critical factor influencing PrEP uptake and adherence (sustained use over time). Studies suggest that individuals who perceive themselves to be at substantial risk are more willing to initiate and continue PrEP.^10,11^

Risk perception is a subjective judgment about the likelihood of a negative occurrence.^12^ It has become a central construct in numerous frameworks designed to predict engagement in health-related behavior.^13–15^ To explore how perceptions of HIV risk shape PrEP uptake and adherence in a pharmacy-based delivery model, this study draws on three complementary theoretical frameworks which include the Tripartite Risk Perception (TRIRISK) model, Protection Motivation Theory (PMT), and the Theory of Planned Behavior (TPB). Each model provides a unique yet overlapping lens through which to examine health decision making. The TRIRISK model classifies risk perception into three components: deliberative (self-asessment of risk exposure and effects), affective (emotional reactions such as fear or anxiety), and experiential (personal or vicarious experiences with risk).^14^ This multidimensional approach enables a more nuanced understanding of how individuals perceive their vulnerability to HIV. Building on this, the PMT focuses on how individuals appraise both the threat of HIV (perceived severity and their vulnerability to it) and their perceived ability to respond to HIV risk exposure through beliefs in PrEP efficacy, their self-efficacy, and the perceived barriers or costs implications.^16^ Lastly, the TPB connects these internal processes to broader social and structural contexts, such as access, stigma, and attitudes toward preventative behavior.^17^ It allows for integration of several known barriers to PrEP use, including side effects, pill burden, anticipated stigma, and service accessibility. It does so by situating them within its key constructs: behavioral beliefs (fear of side effects), normative beliefs (anticipated judgment), and control beliefs (logistical constraints).

Using the TRIRISK model, TPB, and PMT frameworks, this study explored the psychological, social, and structural factors that shape perceptions of HIV risk and shape decisions to engage with and adhere to PrEP. This layered approach allows for a comprehensive understanding of how individuals assess their HIV risk, interpret prevention messaging, and make decisions about initiating and sustaining PrEP use within the context of pharmacy-based delivery. To support conceptual clarity and integration, we will include a schematic analytical diagram to visually represent how each framework contributes to different dimensions of PrEP-related decision making in the results section. The diagram will highlight points of intersection such as risk perception and motivation and demonstrate how the frameworks operate across multiple levels, from individual cognition to social and structural context. This study makes both a theoretical and empirical contribution to the public health literature by integrating these three behavioral sciences models to explore real-world PrEP engagement in a pharmacy-based setting.

## Methods

### Study Design

We employed a phased qualitative design within a pharmacy-based PrEP implementation project. This study was conducted as part of a research centre's PrEP implementation project in South Africa (PPrEPP-SA), which assessed the acceptability and feasibility of pharmacy-based PrEP delivery to improve access, uptake, and adherence. In-depth interviews (IDIs) were conducted pre-implementation (May 2023) and during implementation (April-July 2024) of pharmacy-based PrEP services. The pre-implementation interviews were conducted with participants who had not yet initiated PrEP, capturing their perceptions, motivations, and concerns. During implementation, the interviews were conducted with different participants from the same broader cohort who had initiated PrEP, focusing on their experiences with adherence, evolving motivations, and barriers encountered during use. Although the same individuals were not interviewed at both time points, the sequential interviews with participants at different stages of PrEP engagement provided a temporal perspective on decision making and adherence across the implementation period.

### Study Setting

The study was conducted in 11 community pharmacies across Gauteng and the Western Cape provinces of South Africa. These pharmacies, ranging from small, independently owned outlets to larger national corporate chains, participated in the PPrEPP-SA implementation project. The selected sites were chosen for their accessibility and capacity to provide PrEP services in private, convenient settings where individuals could engage with HIV prevention interventions outside of traditional clinical environments.

### Pharmacy-Based PrEP Delivery Model

Within the pharmacy-based PrEP delivery model, trained pharmacists and pharmacy nurses provided the PrEP services within the pharmacies. These services included HIV testing, PrEP eligibility screening, counseling, and PrEP initiation. Clients who tested HIV-negative and met eligibility criteria were offered oral PrEP and received counseling on adherence, side effects, and HIV risk reduction. Follow-up visits were scheduled to monitor adherence, provide medication refills, and repeat HIV testing in accordance with national PrEP guidelines. Pharmacy staff participating in the project received training to deliver PrEP services and document client visits. PrEP medication and related services were provided as part of the implementation program at no cost to the participants.

### Study Participants and Sampling

Purposive sampling was used to recruit individuals identified as pharmacy clients, defined as persons accessing services at participating pharmacies, who were assessed to be at increased risk of acquiring HIV. Sexual orientation and key population status were not explicitly assessed as part of the eligibility criteria. Risk was determined using a brief screening tool based on self-reported sexual behavior, including indicators such as multiple sexual partners, inconsistent condom use, recent STI diagnosis, or having a partner of unknown or positive HIV status. The screening tool was developed for the implementation project following a review of relevant HIV risk assessment literature and was used to support accurate identification of individuals potentially eligible for PrEP services. Eligible participants were adults aged 18 years and above, who were accessing services at participating pharmacies and identified as being at substantial risk of acquiring HIV, and who provided informed consent. Participants were recruited at 2 separate time points corresponding to the phases of the implementation project. During the pre-implementation phase, individuals who had not yet initiated PrEP were recruited and interviewed to explore their attitudes toward PrEP, perceptions of HIV risk, and reasons for considering or delaying PrEP uptake. During the implementation phase, a separate sample of participants who had initiated PrEP through the pharmacy-based delivery PrEPP-SA project were interviewed. These individuals were drawn from the same broader cohort of pharmacy clients enrolled based on their engagement with pharmacy-based PrEP services during the implementation period. While enrollment in the program indicated initial PrEP initiation, not all participants remained consistently engaged over time. Therefore, these participants were categorized into four groups based on their observed patterns of PrEP use over the 6-month period following initiation: (1) steadily adherent, referring to those who consistently used PrEP as prescribed; (2) not steadily adherent, for those with irregular or inconsistent use; (3) dropped out, for participants who actively discontinued PrEP; and (4) lost to follow up, referring to those who stopped attending follow-up visits, making it unclear whether they continued PrEP use.

### Data Collection and Management

IDIs were conducted at the two time points: pre-implementation and during implementation, using semi-structured guides aligned to the study's conceptual framework. Two different interview guides were adapted for each implementation phase to address phase-= specific research objectives. Overall, the guides included topics on the following: perceptions of individual HIV risk, determinants influencing the initiation or continuation of PrEP, obstacles and enablers of PrEP adherence, the influence of social networks on PrEP decision making (encompassing sexual partners and healthcare providers), and structural elements such as healthcare accessibility and cost.

Questions aligned closely with the study's focused conceptual framework. For example, at pre-implementation, the question “What do you think about our proposed intervention and what makes you feel this way?” reflected participants’ attitudes toward PrEP services and was analytically linked to the TPB. During implementation, the question “Do you consider yourself a suitable candidate for PrEP? Why or why not?” provided insight into participants’ perceived vulnerability to HIV and deliberate risk perception, both central constructs in the TRIRISK model and PMT. The semistructured interview guide was pilot-tested prior to data collection with approximately 10% of the intended study population for readability and understandability.

Interviews were conducted in a private room provided by the pharmacy or via telephone, based on the participant's preferences, and in their chosen language, either in vernacular (any South African language) or English. The interviews lasted for an average of 45 minutes and were audio recorded. The audio recordings were transcribed verbatim and translated into English where necessary for analysis. Author HF independently coded the transcripts and shared them with KEO for review and feedback. Both authors STL-E and MAY conducted a final review of the emerged themes and results to ensure rigor and validity. Reflexivity was maintained throughout the research process, with the research team reflecting on their roles and perspectives during data interpretation.

### Data Analysis

The data analysis followed a deductive thematic analysis approach as outlined by Braun & Clarke.^18^ This deductive framework analysis was guided by PMT, TPB, and the TRIRISK model.^14^ The PMT informed analysis of how participants appraised both the severity of HIV and their perceived capacity to respond through PrEP use, the TPB provided a lens to explore how attitudes, subjective norms, and perceived behavioral control influenced intentions and adherence behavior, while the TRIRISK model was used to explore how participant cognitively, emotionally, and experientially assessed their vulnerability to HIV.

The process began with the familiarization phase to build a comprehensive understanding of participants’ narratives, emotions, and patterns of meaning. This allowed for identification of preliminary insights and areas of thematic relevance. Following this, there was an initial coding phase conducted independently by author HF, reviewed by KEO. Where applicable, STL-E and MAY further revised the initial coding structure.

Codes were applied to highlight aspects of participants’ experiences that aligned with these theoretical constructs, such as Deliberative Risk Perception, Perceived Vulnerability, and Subjective Norms. Illustrative quotes from the transcripts were linked to relevant codes during the analysis process and later selected to exemplify key findings within each theme. These quotes were used to support interpretation and to highlight the participants’ lived experiences.

A comparative analysis of the data collected pre-implementation and during implementation data to explore differences in risk perception and behavior at different points in the program. Transcripts from each phase were coded separately before being compared across shared thematic categories. The analysis was managed using Microsoft Excel 2019 and MAXQDA version 24 software, which facilitated systematic coding, theme development, and data comparison. Thematic saturation was assessed during the analytic process and was considered reached when no new themes or conceptual insights emerged from additional transcripts. Saturation occurred after approximately 15 pre-implementation interviews and approximately 25 interviews in the implementation cohort. To enhance analytic rigor and confirmability, coding decisions and emerging themes were regularly discussed among the coding authors during the analysis process to ensure consistency and credibility of interpretation.

### Credibility/Validity

To ensure the trustworthiness of this study, attention was paid to each dimension of qualitative rigor throughout the research process.^19^ Credibility was supported by conducting interviews in participants’ preferred language and transcribing them verbatim, allowing participants’ own words and meanings to guide the analysis. The inclusion of a diverse client sample, spanning multiple provinces, gender identities, and PrEP experiences, enhanced the transferability of the findings to similar pharmacy-based contexts in South Africa. Dependability was reinforced through the use of semistructured interview guides and a theory-informed coding process, applied consistently across all transcripts. Efforts to ensure confirmability included transparent discussions and documentation of analytic decisions. The reporting of this qualitative study conforms to the consolidated criteria for reporting qualitative research (COREQ) guideline.

## Results

### Participant Characteristics

Pre-implementation, 30 potential PrEP users participated in the IDIs (Table 1). Their ages ranged from 18 to 40 years, with a mean age of approximately 25 years (standard deviation [SD] 6.28). Most participants (22; 73.3%) identified as cisgender women, with 7 identifying as cisgender men, and 1 participant as nonbinary or another gender identity (Table 1). Most participants were Black African (n = 29), with 1 participant identifying as Colored. Participants resided across 4 South African provinces, with most coming from Gauteng (n=14) and Western Cape (n=11) (Table 1).

**Table 1.** Participant Demographics Pre-Implementation and During Implementation.

Category	Pre-Implementation (N = 30)	During Implementation (N = 69)
Age (years)	Mean: 24.6 SD: 6.28	Mean: 31.8 SD: 8.74
	Range: 18-40	Range: 20-59
Gender identity		
Female	22 (73.3%)	34 (49.3%)
Male	7 (23.3%)	35 (50.7%)
Nonbinary	1 (3.3%)	–
Ethnicity		
Black African	29 (96.7%)	66 (95.7%)
Colored	1 (3.3%)	2 (2.9%)
Mixed race	–	1 (1.4%)
Province of residence		
Gauteng	14 (46.7%)	44 (63.8%)
Western Cape	11 (36.7%)	25 (36.2%)
Other	5 (16.6%)	–

During implementation, a total of 69 client participants initiated on PrEP were included in the IDIs (Table 1). Their ages ranged from 20 to 59 years, with a mean age of approximately 32 years (SD = 8.74). In terms of gender identity, 34 participants identified as men, and 35 as women. Most participants identified as Black African (n = 66, 95.7%), with 2 identifying as Colored (2.9%) and 1 as Mixed Race (1.4%). Participants resided in 2 South African provinces: Gauteng (n = 44) and the Western Cape (n = 25).

### Key Themes Mapped Onto the Theoretical Frameworks

The analysis revealed a range of interrelated themes that map onto the theoretical frameworks guiding this study. These themes emerged from both the pre-implementation and during implementation interviews, offering a layered understanding of how individuals conceptualize their HIV risk, weigh the costs and benefits of PrEP, and make decisions about initiation, adherence, or discontinuation. The themes are systematically categorized by the theoretical models and key theme areas, as shown in Table 2.

**Table 2.** Key Themes Categorized by Theoretical Framework.

Theoretical Framework	Theme/Construct	Subtheme/Focus Area	Explanation
TRIRISK model	Deliberative risk perception	Awareness of risk	Participants’ conscious evaluation of their likelihood of acquiring HIV
	Affective risk perception	Emotions relating to risk	Feelings such as fear, anxiety, or indifference associated with perceived risk
	Experiential risk perception	Experiences with risk	Judgments based on past experiences or observed events involving HIV risk
Protection Motivation Theory	Threat appraisal	Perceived severity	Beliefs about the seriousness of acquiring HIV and its consequences
		Perceived vulnerability	How likely participants felt they were to contract HIV
		Fear or anxiety	Emotional responses to perceived HIV threat (eg, fear of infection)
	Coping appraisal	Response efficacy	Beliefs about PrEP's effectiveness in preventing HIV
		Self-efficacy	Confidence in one's ability to access and consistently take PrEP
		Response costs	Perceived barriers, such as side effects, stigma, or cost of PrEP
Theory of Planned Behavior	Attitudes	Beliefs and feelings about PrEP use	Positive or negative evaluation of using PrEP for HIV prevention
	Subjective norms	Social influence and perceived expectations	Perceptions of how significant others (eg, peers, partners) view PrEP use
	Perceived behavioral control	Ease or difficulty in using PrEP	Perceived ability to start and maintain PrEP given personal or structural factors.

Abbreviations: HIV, human immunodeficiency virus; PrEP, pre-exposure prophylaxis; TRIRISK: Tripartite Risk Perception.

## Risk Perception Based on the TRIRISK model

**Figure 1.**
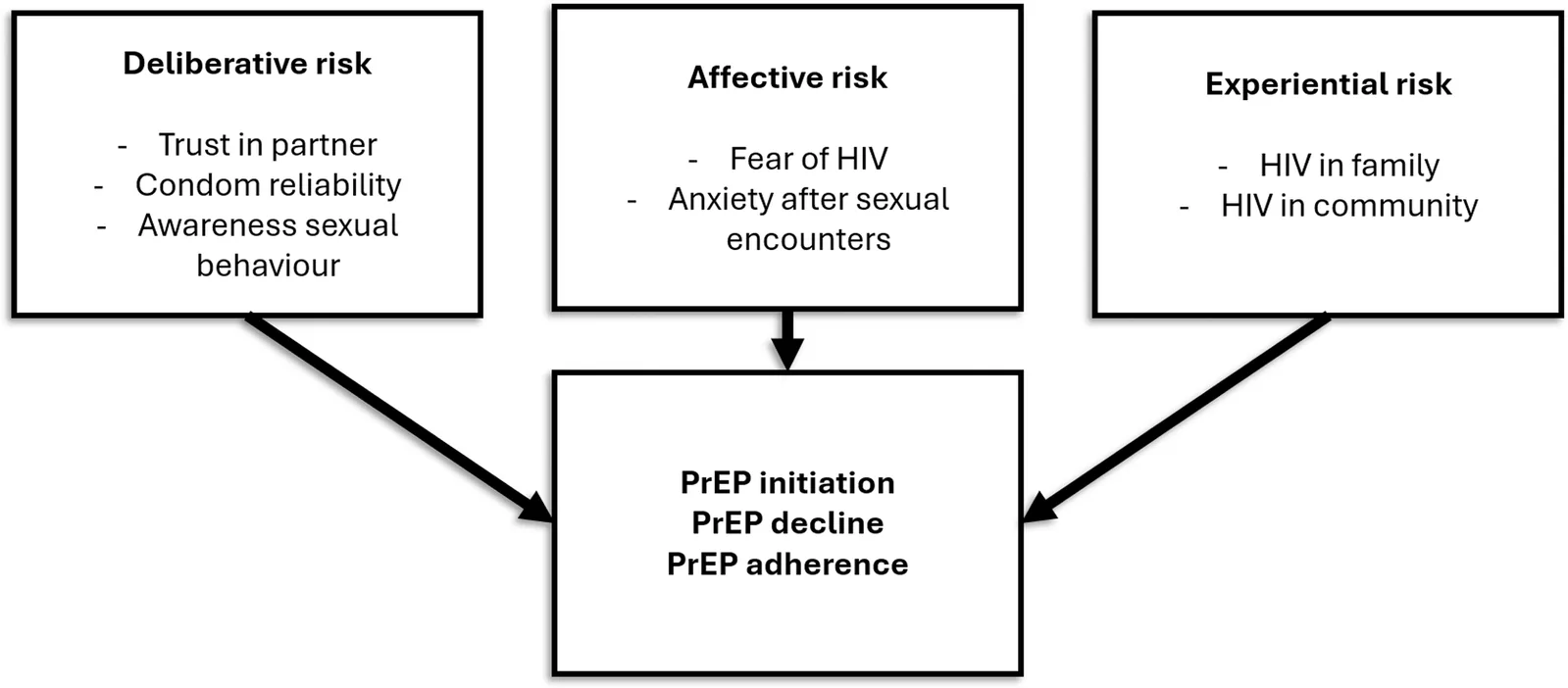
TRIRISK model and PrEP use. Abbreviations: PrEP, pre-exposure prophylaxis; TRIRISK: Tripartite Risk Perception.

Participants’ decisions around PrEP were shaped by rational judgments, emotional responses, and personal experiences as described in the TRIRISK framework^14^ and depicted in Figure 1. Some assessed their HIV risk based on partner behaviors or sexual activity, leading them to either initiate PrEP or dismiss the need for it. Emotional drivers such as fear of infection or past trauma also played a role, sometimes motivating uptake, but at times causing avoidance. For others, direct experiences with illness, sexually transmitted infection (STI) diagnoses, or seeing loved ones affected by HIV made their risk feel more real, reinforcing the need for protection. We now explore each of these drivers in turn.

### Deliberative Risk Perception

Participants often assessed their HIV risk based on perceived rational factors such as trust in monogamy, prior HIV testing, or their own sexual behaviors. Many described using cognitive reasoning to justify their decisions about protection and prevention. However, among those interviewed pre-implementation, there was also recognition of the unpredictability of risk, particularly around condom reliability and partner honesty. As one participant among participants interviewed pre-implementation explained, highlighting the unpredictability of condom use and partner honesty:

Sometimes you can't always trust your partner, or maybe the condom bursts or anything like that. Some people aren't upfront about their status so you will find yourself not being protected. (34 years—Female, Pre-implementation)

During implementation, another participant reflected on their inconsistent use of condoms, tying their PrEP adherence to these behaviors:

So, I like, I've been very reckless, you know. I mean like not using protection. Yeah, that's what I mean. Close to never… Okay, I only use condoms when like, I don't like, know, the, what's this thing? If I'm taking the, if I'm not taking the pills, I'll use condoms. Okay. If I'm not, I'll probably not use the condoms. Before. Like before I started the programme. (22 years—Male, During implementation, Not Steadily Adherent)

Some participants justified noninitiation of PrEP based on a belief in mutual monogamy and trust. One pre-implementation participant expressed confidence in their partner's fidelity and viewed their own loyalty as sufficient protection:

And I did consider it[PrEP], but because I trust my partner and I can see I’m loyal. I don't go around sleeping, so I don't think I need it because if I trust my partner and she’ll be the only person I’m sleeping with. If we’re both HIV negative, then I’m safe. That's why I didn't get the medicine education. (18 years—Male, Pre-implementation)

However, trust-based reasoning was often challenged by later experiences. In contrast to earlier beliefs, a during implementation participant disclosed:

I don't know. Because me and him have a very transparent, very open relationship with each other. You know, so I, where he's concerned, I feel like I don't have anything to worry about. (34 years—Female, During implementation, Dropped Out)

Such trust-based assessments were not always accurate, though. At during implementation, another participant described discovering their long-term partner's infidelity and reflected on the unexpected risk this posed. This experience led them to reassess their earlier belief in being in a low-risk situation:

So, I just found out that my long-term partner is having an affair with another person and the person was thinking they are pregnant, and it means they were not using protection, so I never really know how the other person is regarding their health and all of that. (24 years—Female, During implementation, Dropped Out)

### Affective Risk Perception

Participants evaluated the emotional dimensions of their HIV vulnerability, such as fear, anxiety, or trauma-related responses. These feelings often amplified a sense of urgency, influencing the intention to start PrEP. For some participants, fear was a central motivator, shaped not only by risk behavior but also by the psychological burden of past decisions. One participant from the during implementation interviews reflected on the intense anxiety he experienced around the possibility of being infected with HIV, describing a deep, personal fear of the virus. He explained that his decision to take PrEP was rooted in a desire to avoid one of the 2 things he fears most in life: jail and HIV:

Yeah, the reason why I'm taking PrEP, the main reason why I take PrEP is just to protect myself from getting infected with HIV …I’m scared of two things in this life. One is jail and the second is sickness. (37 years—Male, During implementation, Not steadily adherent).

When the interviewer sought clarification, he emphasized that while he wasn't afraid of general illness, HIV was the one condition that truly terrified him. He first heard about PrEP from a friend and decided to give it a try.

Similar to the views, pre-implementation participants, during implementation participants described how an encounter with person living with HIV led to lasting trust issues and emotional detachment from health-seeking behaviors. Their emotional reaction became a barrier as reflected in the quote below:

Since the incident of the guy, of that scenario[having sex with HIV-positive person who she forced to use protection], I have been having trust issues… Even if we are using a condom, I will never get satisfied that you are really HIV negative. So, I think somehow, I got affected by that to such an extent that I don't want anything to do with my health. (27 years—Female, During implementation, LTFU)

### Experimental Risk Perception

Lived or witnessed events related to HIV sometimes served as a turning point, leading participants to re-evaluate their susceptibility and act upon it. For some, the broader community impact of HIV was a foundational reason for embracing PrEP. One participant from pre-implementation interview referenced the high mortality rates from HIV in their community and saw PrEP as a necessary form of proactive protection:

Because no, we have died a lot as a black nation because of HIV. So, I think this PrEP will really help a lot with decreasing it. This is why it's no longer that much because the government has tried. But let us protect ourselves. We must prevent it and not treat it as though ‘it's already there anyway’. (28 years—Female, Pre-implementation)

In a similar vein, a during implementation participant who lost siblings to HIV described how witnessing the decline and death of 2 sisters from HIV motivated them to take action. Their fear of unknowingly contracting the virus also encouraged them to continue using PrEP:

And then I had seen the HIV effect from my sisters, how they ended up like dying, do you understand? So that is why I was encouraged to not end up like them… Like I said what really encouraged me from the beginning was the thing with my sisters … I had a fear that maybe I might have it … But then by God's grace, I found out I didn't have it. So that's why I was encouraged to go on taking PrEP. (21 years—Female, During implementation, Dropped Out)

## Protection Motivation Theory

**Figure 2.**
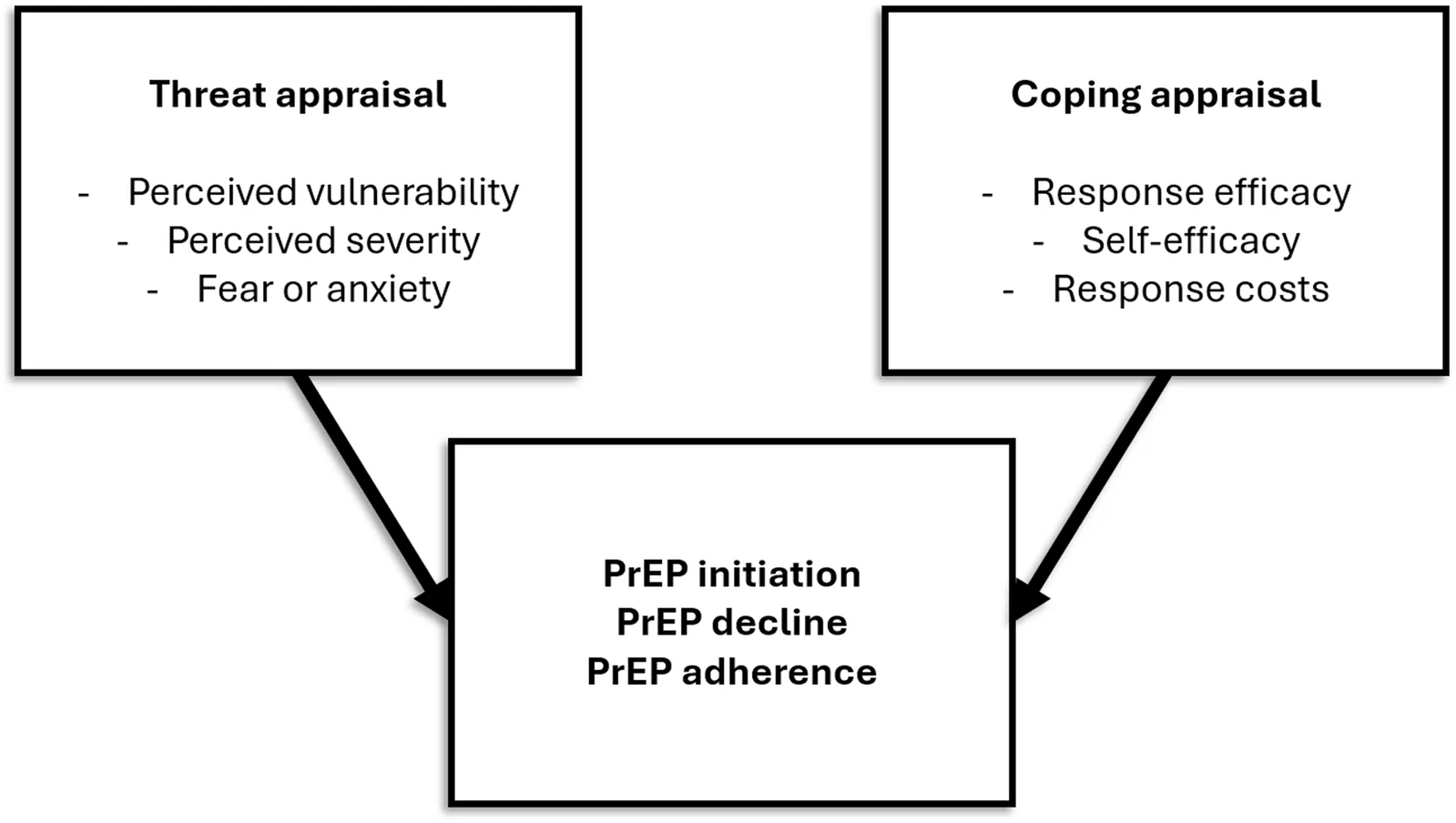
Influence PMT on PrEP use. Abbreviation: PMT, Protection Motivation Theory; PrEP, pre-exposure prophylaxis.

Motivations to start or continue PrEP were also shaped by how participants perceived the threat of HIV and their ability to manage risk. Most participants viewed HIV as a serious danger and felt vulnerable due to their own or partners’ behaviors, while others weighed their ability to adhere to PrEP. Confidence in PrEP, access through pharmacies, and trust in the healthcare setting often strengthened uptake, whereas doubts, forgetfulness, or lifestyle disruptions posed challenges. These dynamics are explored in more depth in the sections below and visualized in Figure 2.

### Threat Appraisal

Under PMT, threat appraisal reflects how individuals assess both the severity of HIV infection, their own vulnerability to contracting it, and their fears and anxiety surrounding it. Across interviews, these dimensions played varying roles in shaping decisions to start or continue PrEP: perceived severity, perceived vulnerability and fear or anxiety.

### Perceived Severity

HIV was recognized as a life-altering condition with consequences that extended beyond individual health to affect entire communities. This collective awareness shaped how some framed PrEP as a necessary protective measure. A pre-implementation in 2023 participant emphasized this communal sense of urgency:

I would say we must take PrEP pill because at the end of the day, it's our lives, and we have to save our lives because now people are dying … let's just take PrEP you see, because the disease is there, HIV and AIDS, so we must take care of our bodies and our lives. (23 years—Female, Pre-implementation)

### Perceived Vulnerability

With regard to perceived vulnerability, participants viewed their lifestyles, multiple partners, and inconsistent condom use as increasing their HIV risk, thus justifying continued PrEP use. A during implementation participant reflected on their sexual behaviors and relationship dynamics:

Ah, it's a lot, it's a lot of people, it's a lot. It's not one person[sexual partners] it's just a lot. Like I wouldn't say it's … It could be anyone that's easy going …Well, sometimes there is protection and with the other two[stable sexual partners], there is no protection. (31 years—Male, During implementation, Steadily Adherent)

### Fear or Anxiety

In the fear and anxiety domain, participants referenced unpredictable situations and a general sense of vulnerability to unforeseen events. Such expressions illustrate how risk was sometimes understood not only through deliberate reflection on behavior, but also through broader concerns about the unpredictability of life and external threats:

Things happen. You will never know what might happen. People are raped out there. (39 years—Female, During implementation, LTFU)

## Coping Appraisal

Participants’ decisions to initiate and continue PrEP were also shaped by their confidence in their ability to manage the perceived risk of HIV exposure effectively. This included confidence in PrEP's effectiveness, their own ability to adhere to the regimen, and an assessment of the barriers or costs involved.

### Response Efficacy

Belief in the effectiveness of PrEP emerged as a strong motivator for continued use. This is illustrated in the experience from this participant at during implementation, who initially doubted the medication developed trust in it over time:

At first, I was skeptical about it… you're taking the medication, however you don't trust it. So, I was taking PEP and also using a condom … But yeah, and then ever since I started using PrEP, I had no issues about my status whatsoever. No, I'm not even scared to take a self-test … because I trust the pill so far. (24 years—Male, During implementation)

### Self-Efficacy

Despite recognizing PrEP's benefits, participants expressed varying levels of confidence in their ability to take it consistently. Forgetfulness and competing priorities were common barriers. Even when strategies like setting alarms were used, adherence could be compromised by everyday disruptions, leading to occasional missed doses:

There were times I forgot. I set an alarm. There were times when the alarm would go off and I wouldn't be home. When I get home, I’d forget to take it and think about it when I’m already asleep … Let's say one to three[times forgotten per month]. (23 years—Female, During implementation, Not Steadily Adherent)

### Response Costs

Participants compared pharmacy-based PrEP services to those offered at public clinics, frequently praising the pharmacy model for its privacy, convenience, and efficiency as opposed to clinics:

Some nurses at the clinics aren't really confidential … it[the pharmacy] gives you privacy, you don't have to stand in long queues only to be treated very badly – bad services. So yah … It's much better than going to the clinic. It's much more efficient … it's a safe space. (20 years—Female, Pre-implementation)

## Theory of Planned Behavior

**Figure 3.**
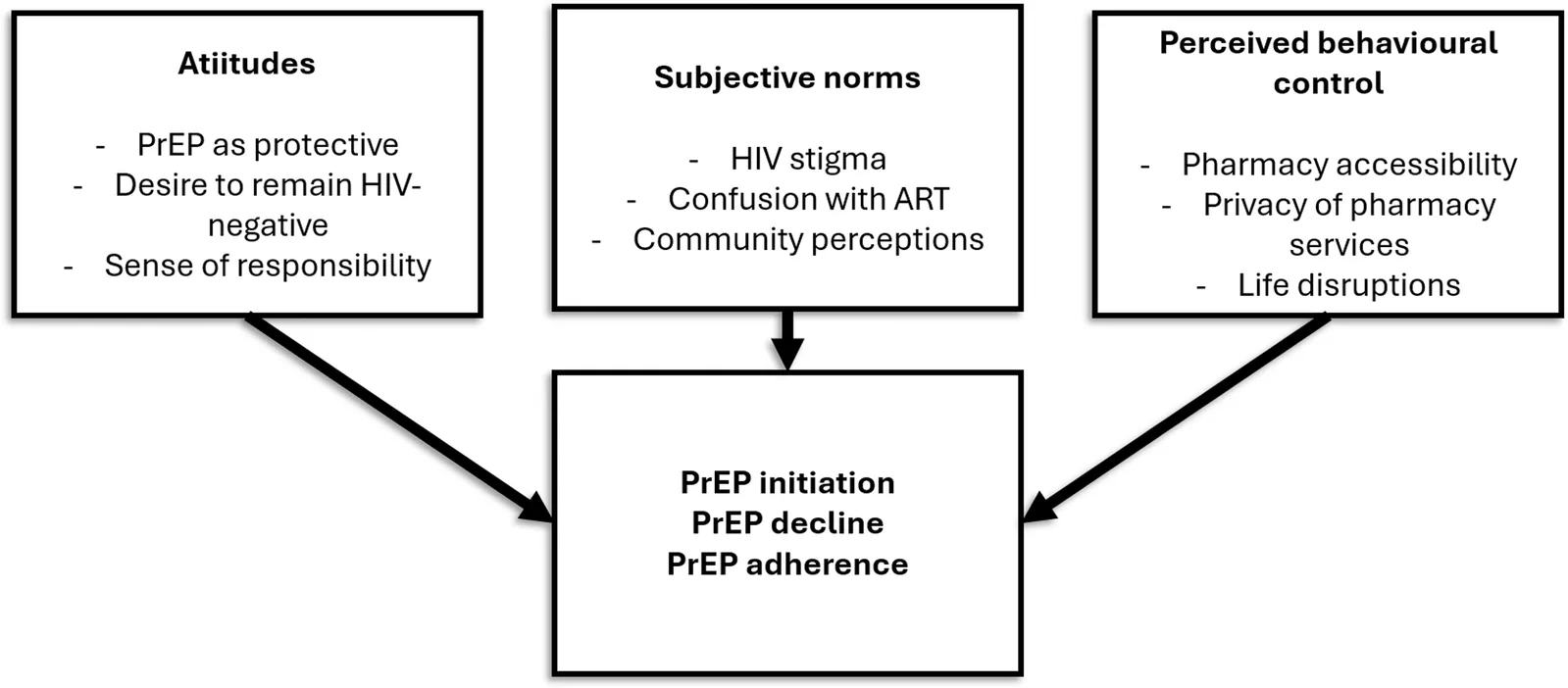
Influence TPB on PrEP use. Abbreviations: PrEP, pre-exposure prophylaxis; TPB, Theory of Planned Behavior.

Participants’ decisions were also shaped by attitudes, perceived norms, and their sense of control. While attitudes were mostly positive across time points, subjective norms, and structural challenges continued to complicate adherence. Furthermore, some participants saw PrEP as empowering and health-enhancing, whereas others felt conflicted due to stigma or concerns about daily adherence. Social support, from peers, partners, or healthcare providers played a key role, as did individuals’ confidence in managing the routine of PrEP use. The interplay of these factors is unpacked in the sections that follow and illustrated in Figure 3.

### Attitudes

Participants expressed overwhelmingly positive attitudes toward PrEP, grounded in personal health goals and a sense of responsibility toward others. These attitudes were not necessarily linked to perceptions of high-risk behavior but reflected a broader desire to maintain wellness and avoid infection. Participants emphasized prevention as part of protecting not just themselves, but their children and the broader community through practices like blood donation:

Yes. I felt like I just wanted to stay HIV free. So, I must look after my health, because normally I do donate blood. So, the people that I donate blood to, I don't want to give people my HIV. So I decided that for my health, my kids. So that I can take PrEP to prevent myself from being infected. That's what I did. (39 years—Female, During implementation, Steadily Adherent)

Just like the during implementation participant view, pre-implementation participants emphasized the importance of viewing PrEP as a proactive health decision, especially in light of the broader HIV landscape in South Africa. Rather than associating PrEP use with specific identities or behaviors, they framed it as a general health priority particularly for young people:

I think looking at the statistics of South Africa, many of the youth are infected with HIV so taking a step as to protecting your health you should have a PrEP. So, protecting your health should be your first priority. (20 years—Female, Pre-implementation)

### Subjective Norms

Subjective norms played a noteworthy role in both facilitating and inhibiting PrEP engagement. These social pressures often intersected with stigma, secrecy, and the need for social approval in relationships or communities.

Social perceptions and community attitudes played a key role in how participants navigated conversations about PrEP. A recurring concern was the community's widespread confusion between PrEP and ART, the medication taken by people already living with HIV. This misunderstanding often led to assumptions that PrEP users were HIV-positive, reinforcing stigma. One pre-implementation participant reflected on this challenge:

People … they don't know, maybe you helped someone and then you get infected … Because it's related to HIV. Like people they don't have knowledge of what PrEP is, and the only thing that they know when you’re having PrEP, you’re positive. (23 years—Female, Pre-implementation)

Such misconceptions made it difficult for participants to talk openly about their PrEP use, especially with intimate partners. Some feared that disclosure could trigger suspicion or accusations of infidelity, rather than being seen as a proactive health decision. One participant shared that while conversations with friends felt more comfortable, bringing up the topic with partners carried the risk of judgment:

Yes, mostly I talk about PrEP to my friends. I have not had the guts to talk about it with my partners … Partners turn to be judgemental on the PrEP. You have to come up with the reason why did you decide to take PrEP. (43 years—Male, During implementation, Steadily Adherent)

### Perceived Behavioral Control

Participants’ ability to consistently engage with PrEP services was shaped by both the accessibility of pharmacy-based distribution and their personal life circumstances. Many participants praised the convenience of pharmacy access, noting that it reduced time burdens and normalized PrEP collection by integrating it into daily routines. One participant emphasized how the widespread presence of pharmacy chains like Clicks increased ease of access:

So, I think it's a great idea[PrEP in pharmacy] because it makes it more available to everyone and it's inclusive. Because it's so convenient like pharmacies like Clicks are scattered like everywhere … so the convenience of just being at the mall or something and then going to a pharmacy to get PrEP, it's also less time consuming … (18 years—Female, Pre-implementation)

However, perceived control was not uniform. Structural barriers such as financial instability, caregiving responsibilities, and lack of transport constrained the ability of some participants to maintain access. One participant described how a series of personal hardships, relocation, and financial strain disrupted their routine and ultimately led to discontinuation. This contrasting experience compared to the view by participants’ pre-implementation is illustrated below.

After September my grandfather passed away, and I had to go down to the village… There is no work. It was difficult to go to the pharmacy without money … In January my mother got sick … I stopped many things, till her passing away … Transport is the problem with us[PrEP access]. (21 years—Female, During implementation, Dropped Out)

## Triangulation of Findings

Triangulated insights from the data are summarized in Table 3. While participants differed across time points, the analysis identifies both continuity and variation in subthemes that emerged during different phases of program implementation. For example, deliberative assessments of risk, often grounded in trust, were more commonly expressed by participants interviewed prior to implementation. In contrast, participants who had initiated PrEP during implementation more frequently described heightened awareness of risk following experiences of infidelity. Affective responses such as anxiety were also notable and appeared more closely tied to lived experience among the PrEP users. Although PrEP was consistently viewed as effective across both groups, participants initiated on PrEP more often reported concerns about adherence and continuity, often linked to life disruptions such as travel. Pharmacy-based delivery models enhanced perceptions of control and privacy at both time points, though practical barriers such as transport and financial constraints, remained a challenge. These patterns reflect the dynamic interplay between individual perceptions, lived realities, and structural conditions shaping PrEP engagement, while also acknowledging the limitations of comparing distinct participant groups across different time points.

**Table 3.** Triangulation of Findings Across Frameworks and Time Points.

Theoretical Framework	Theme	Key Topics From Pre-Implementation (IDI Quotes)	Key Topics From During Implementation (IDI Quotes)	Narration of Patterns Across Time Points
TRIRISK	Deliberative	Trust in monogamy	Risk awareness shaped by infidelity experiences	Both groups showed deliberate risk assessments, though participants who initiated PrEP during implementation more often described disruptions to trust based on personal experiences
	Affective	Anxiety about testing, fear of HIV	HIV-related trauma and emotional burden	Affective responses were present across time points; among those who initiated PrEP during implementation, emotions were more often grounded in lived experience
	Experiential	Community/family awareness of HIV	Personal and family events influencing perception	Across both groups, experiential exposure to HIV risks (eg, illness or loss) influenced motivation to use PrEP
PMT	Perceived vulnerability	HIV framed as deadly and socially pervasive	HIV viewed as serious and life-threatening	Perceived vulnerability remained high overall, with PrEP users often linking it to more immediate personal or relational contexts
	Perceived severity	Concerns about multiple partners, low condom use	Realizations about partner infidelity	Severity concerns were common across time points; participants who initiated PrEP more often cited concrete relational breaches
	Fear or anxiety	General fear of HIV or sexual violence	Fear shaped by real events and uncertainty	While both groups expressed fear, those who initiated PrEP during implementation more often described fears rooted in recent personal experience
	Response efficacy	Trust in PrEP as protective	Strong belief in PrEP's effectiveness	Belief in PrEP's effectiveness was evident across groups; for some who had initiated PrEP, this belief appeared reinforced through use
	Self-efficacy	Optimism about starting and sticking to PrEP	Challenges due to disrupted routines or forgetfulness	Perceived self-efficacy varied; participants who had started PrEP during implementation often described more frequent adherence-related challenges
	Response costs	Clinics seen as stigmatizing; pharmacies viewed more positively	Pharmacies preferred, but logistical and cost barriers emerged	Pharmacies were viewed as enabling, but participants who had initiated PrEP during implementation more commonly raised concerns about transport and affordability
TPB	Attitudes	PrEP seen as empowerment and health-conscious choice	PrEP framed as self-care and relational responsibility	Positive attitudes persisted across groups; those who initiated PrEP during implementation more often framed it as part of a shared or relational responsibility
	Subjective norms	Fear of being judged; confusion with ART	Persistent stigma, but some increase in acceptance	Stigma remained a concern, though participants who initiated PrEP during implementation sometimes noted growing social familiarity
	Perceived behavioral control	Pharmacy access increased sense of agency	Life disruptions and financial strain reduced perceived control	While pharmacy-based models supported agency, participants who had initiated PrEP more frequently described external challenges limiting access and continuity

Abbreviations: ART, antiretroviral therapy; HIV, human immunodeficiency virus; IDIs, in-depth interviews; PMT, Protection Motivation Theory; PrEP, pre-exposure prophylaxis; TPB, Theory of Planned Behavior; TRIRISK: Tripartite Risk Perception.

## Discussion

This study set out to understand the nuanced, subjective aspects that influence PrEP uptake and adherence through the TRIRISK model, PMT, and TPB. Overall, findings highlight that risk perception plays a pivotal role in shaping decisions to initiate, continue, or discontinue pharmacy-based PrEP programs. Participants’ perceptions of HIV risk, coupled with emotional responses and social influences, guided their PrEP-related behaviors. Structural factors, including access to healthcare, affordability, and stigma, were notable influencers of long-term adherence to PrEP. This study offers a unique contribution to the literature by synthesizing 3 complementary theoretical frameworks the TRIRISK model, PMT, and TPB to explore PrEP decision making within a pharmacy-based PrEP delivery model. This multitheoretical approach provides a more nuanced understanding of the cognitive, emotional, social, and structural determinants that shape HIV prevention behavior. While these models have been applied individually in prior research including the use of TPB to explore PrEP uptake among breastfeeding mothers in Zambia,^20,21^ PMT to assess adherence to COVID-19 prevention measures,^22^ and the TRIRISK model in studies on HIV risk in Brazil and in non-HIV domains such as smoking cessation^23^ and healthcare safety culture,^24,25^ their application within pharmacy-based HIV prevention settings, particularly in African contexts, remains limited. To our knowledge, this is the first study to integrate these three frameworks in a real-world evaluation of pharmacy-based PrEP delivery. This combined approach offers deeper and more comprehensive insights than prior studies that examined each model in isolation.

In relation to the TRIRISK and PMT frameworks, participants’ perceptions of HIV risk were shaped by a dynamic interplay of rational evaluation, emotional response, and lived experience. Among those interviewed prior to implementation, many described deliberative assessments rooted in trust and assumptions of monogamy, which often led to an underestimation of personal risk. In contrast, participants who had initiated PrEP during implementation more frequently reported that such assumptions had been challenged by experiences such as partner infidelity or STI diagnoses, which contributed to greater risk awareness and shifts in behavior. Perceived vulnerability was often discussed in more abstract or hypothetical terms by participants, whereas those who had begun PrEP more commonly described vulnerability in concrete and personally validated ways. Perceptions of severity were consistently high across both time points, with HIV viewed as a serious and ongoing threat supporting a strong threat appraisal across the sample. Emotional responses such as fear, trauma, and anxiety were evident throughout, though these were typically described in more generalized terms pre-implementation. Among participants who initiated PrEP during implementation, emotional responses were more often anchored in specific and recent lived experiences. This shift from hypothetical to lived vulnerability aligns with findings of Chimukuche et al (2025)^26^ where participants’ understanding of HIV risk evolved over time through direct or vicarious experiences. Similar patterns have been observed in studies examining HIV risk perception, which show that experiential and affective perceptions of risk can strongly influence preventive behavior and willingness to adopt protective strategies such as PrEP.^11,14^ In this study, as risk became more personal and emotionally charged, motivation for PrEP use and adherence increased, shaped by a growing recognition that HIV was not only a societal issue but an immediate and personal concern. Many participants trusted in PrEP's biomedical effectiveness, yet daily adherence was often disrupted by forgetfulness, pointing to the gap between perceived efficacy and actual behavioral capability.

Personal or familial experiences with HIV loss further reinforced the seriousness of the threat, motivating PrEP initiation and continuation for some. According to Singleton et al,^27^ fidelity has historically been promoted through loss-framed messages that emphasize the dangers of infidelity and nonmonogamy in the context of HIV. These negative outcomes include risks for HIV transmission, familial wellbeing, moral character, or a combination of these, and this evidence is consistent with the demonstrated findings from this study on how fidelity influences HIV risk perception further motivating PrEP uptake.^27^ Furthermore, another study conducted among women in parts of Kenya and South Africa confirms the findings of this study, which found that motivators for perceived HIV risk and PrEP uptake were influenced by experiences such as having HIV-positive family members or losing family members due to HIV complications. Participants in the study feared contracting HIV “from” or “like” their family members, as identified in this study.^28^ These evidences also reflect how PMT framework reveals participants’ perceived threats and coping strategies.

Findings based on the TPB model suggest that stigma and misinformation, particularly the confusion between PrEP and ART, plays a role in PrEP uptake and disclosure especially with most people fearing being identified as HIV-positive. This stigma, which is linked to subjective norms, made disclosure difficult, especially in intimate relationships. While social attitudes improved slightly over time, making PrEP more acceptable, practical issues such as low socioeconomic position was noted to affect PrEP uptake adherence despite the ease of accessibility and convenience the pharmacy setting confer. Thus, even with positive perceptions, structural issues could negatively affect PrEP uptake and persistence. Drawing on these findings, reducing PrEP-related stigma emerges as a critical priority for improving access and uptake. When PrEP was first introduced globally by the WHO in 2015,^29^ numerous studies identified stigma as a key barrier often intensified by individuals’ prior experiences of discrimination in healthcare settings. These encounters heightened sensitivity to PrEP stigma, making people less likely to seek out or remain engaged in care. Despite clear and consistent evidence highlighting the harmful impact of stigma on health-seeking behavior,^30^ it is concerning that stigma continues to persist as a major obstacle. This ongoing challenge underscores the urgent need for interventions that directly confront and dismantle stigma in both community and healthcare contexts to ensure more equitable access to PrEP.

While PrEP was generally seen as proactive, health-affirming step, social perceptions, and structural enablers played a pivotal role in whether participants maintained adherence. Several during implementation participants cited the convenience, privacy, and efficiency of pharmacy-based PrEP models as supportive of ongoing use. However, many still faced challenges maintaining consistent adherence due to life disruptions such as relocation, changing work schedules, and caregiving responsibilities, making it difficult to prioritize daily pill-taking or attend follow-up visits. These structural factors often outweighed individual motivation and risk awareness and caused discontinuation of PrEP use. ^31^^31^

The triangulation of findings across the three frameworks pre-implementation and during implementation revealed a dynamic interaction between individual cognition, emotional experience, and contextual constraints, further enhancing the validity of the study′s findings.^32^ While perceptions of HIV risk and the perceived relevance of PrEP remained relatively consistent, emotional responses, and adherence challenges differed at the two time points. Notable differences were visible between individuals interviewed pre-implementation and those who were initiated on PrEP and interviewed during implementation (6 months and longer into the study timeline). Personal experiences such as partner infidelity, risk sexually behavior, death of loved one from HIV related complications were seen to trigger a reassessment of HIV risk. As a result, emotional responses at during implementation were more nuanced, moving from generalized fear to context-specific anxieties grounded in lived realities.

The integration of the three frameworks offered complementary and layered insights. While PMT helped explain how participants transitioned from perceived threat to protective motivation, TPB clarified the role of subjective norms and perceived control in shaping behavior. The TRIRISK model enriched this understanding by capturing the evolution of dimensions of risk. This emotional evolution shaped both attitudes toward PrEP and decisions related to its initiation and sustained use. By combining these frameworks, we were able to explore not only what participants thought about their HIV risk, but also how they felt, how they acted, and how these processes were shaped by dynamic social and structural conditions. In summary, while knowledge and positive attitudes toward PrEP may persist over time, within our study, lived experience and structural conditions appeared to influence whether PrEP adherence is persistent or discontinued. These findings highlight the importance of adaptable, empathetic, and context-sensitive PrEP delivery models that are responsive to the changing realities of users.

### Strengths and Limitations

The study offers valuable insights; however, the findings may not be generalizable to all regions of South Africa, particularly to key populations such as men who have sex with men (MSM) and individuals residing in rural areas. While it is possible that some participants may have belonged to key populations, the study did not explicitly collect data on sexual orientation or key population status, as eligibility criteria were based primarily on age and self-reported sexual risk behaviors. As a result, the analysis was not designed to examine the unique social, behavioral, and structural contexts that may shape PrEP use among MSM or other key populations. Because the participating pharmacies were situated in urban settings, the sample primarily reflects the experiences of urban and peri-urban pharmacy-based PrEP users within a specific implementation project. As a result, populations outside this programmatic reach including individuals unaware of PrEP, those facing logistical or systemic barriers to access, or those living in more remote provinces were not captured in this analysis.

Furthermore, participants interviewed during the pre-implementation phase were not the same individuals interviewed during the implementation phase, which limits the ability to draw direct longitudinal conclusions. Consequently, the findings should be interpreted as reflecting perspectives from different participants at distinct stages of program delivery rather than direct longitudinal changes over time. Differences observed between the two phases may therefore represent variation in individual experiences rather than true temporal shifts in perceptions or behavior, and these limitations should be considered by future studies.

In addition, there was conceptual overlap across the behavioral frameworks used. For instance, some logistical or relational barriers were interpreted both as high response costs within PMT framework and as low perceived behavioral control within TPB. Similarly, perceived vulnerability sometimes intersected with deliberative reasoning within the TRIRISK model, making it difficult to distinctly categorize participant responses. This analytical overlap reflects the complexity of lived experiences, which do not always align neatly with theoretical constructs. While the use of multiple frameworks added depth, it also introduced some interpretive overlap that should be considered when drawing conclusions.

Despite these limitations, the study provides important insights into how psychological, social, and structural factors influence PrEP uptake and adherence within a real-world pharmacy-based delivery model. The integration of multiple behavioral frameworks also enabled a more comprehensive understanding of how individuals perceive HIV risk and navigate decisions about initiating and sustaining PrEP use.

## Conclusion

Risk perception can play a critical role in the uptake and adherence to pharmacy-based PrEP programs among high-risk South Africans. Understanding the psychological, social, and structural factors shaping how individuals perceive their HIV risk is essential for tailoring health interventions that improve PrEP uptake and long-term use. The application of integrated behavioral frameworks such as the TRIRISK model, PMT, and TPB provides a valuable lens for designing context-sensitive interventions. These frameworks can help to explain how individuals assess HIV risk, evaluate their coping capacity, and navigate interpersonal and structural influences when deciding whether to initiate or remain on PrEP.

This study offers several practical implications for policy and program design. Pharmacy-based PrEP delivery models should be complemented by public health messaging that positions PrEP as a proactive and responsible health choice. Misconceptions equating PrEP with HIV treatment or promiscuity must be challenged through stigma-sensitive communication. Reframing PrEP as a component of routine self-care may increase social acceptance and reduce judgment from peers, partners, and healthcare providers. To improve adherence, interventions should enhance individuals’ coping ability and behavioral control. This includes the use of practical tools (eg, reminders or adherence apps), decentralized or mobile refill systems, and service delivery models that accommodate life disruptions such as relocation, caregiving, or financial instability. Ensuring flexibility and autonomy in access points can help maintain continuity of care and reduce drop-off.

For policy makers, these findings highlight the need to integrate behavioral science into national HIV prevention strategies. PrEP programs should go beyond biomedical provision and consider emotional, social, and logistical factors influencing decision making. This requires cross-sector collaboration between pharmacy chains, public health departments, and community organizations. Future research should extend the use of integrated behavioral models to inform adaptive interventions that evolve alongside users’ needs. Longitudinal studies are especially valuable for capturing how motivation, perceived risk, and adherence fluctuate over time. Embedding behavioral frameworks in the design, delivery, and evaluation of PrEP services will ensure they remain responsive, inclusive, and effective across diverse settings.

## Supplemental Material

sj-pdf-1-jia-10.1177_23259582261447318 - Supplemental material for Risk Perception and Its Influence on Pre-Exposure Prophylaxis (PrEP) Uptake and Adherence Among Pharmacy-Based PrEP Users in South Africa: A Qualitative Study Supplemental material, sj-pdf-1-jia-10.1177_23259582261447318 for Risk Perception and Its Influence on Pre-Exposure Prophylaxis (PrEP) Uptake and Adherence Among Pharmacy-Based PrEP Users in South Africa: A Qualitative Study by Hugo Ferrero, Kelechi Elizabeth Oladimeji, Mara Aileen Yerkes, Athini Nyatela, Cheryl Hendrickson, Angela Tembo and Samanta Tresha Lalla-Edward in Journal of the International Association of Providers of AIDS Care (JIAPAC)

sj-pdf-2-jia-10.1177_23259582261447318 - Supplemental material for Risk Perception and Its Influence on Pre-Exposure Prophylaxis (PrEP) Uptake and Adherence Among Pharmacy-Based PrEP Users in South Africa: A Qualitative Study Supplemental material, sj-pdf-2-jia-10.1177_23259582261447318 for Risk Perception and Its Influence on Pre-Exposure Prophylaxis (PrEP) Uptake and Adherence Among Pharmacy-Based PrEP Users in South Africa: A Qualitative Study by Hugo Ferrero, Kelechi Elizabeth Oladimeji, Mara Aileen Yerkes, Athini Nyatela, Cheryl Hendrickson, Angela Tembo and Samanta Tresha Lalla-Edward in Journal of the International Association of Providers of AIDS Care (JIAPAC)

sj-pdf-3-jia-10.1177_23259582261447318 - Supplemental material for Risk Perception and Its Influence on Pre-Exposure Prophylaxis (PrEP) Uptake and Adherence Among Pharmacy-Based PrEP Users in South Africa: A Qualitative Study Supplemental material, sj-pdf-3-jia-10.1177_23259582261447318 for Risk Perception and Its Influence on Pre-Exposure Prophylaxis (PrEP) Uptake and Adherence Among Pharmacy-Based PrEP Users in South Africa: A Qualitative Study by Hugo Ferrero, Kelechi Elizabeth Oladimeji, Mara Aileen Yerkes, Athini Nyatela, Cheryl Hendrickson, Angela Tembo and Samanta Tresha Lalla-Edward in Journal of the International Association of Providers of AIDS Care (JIAPAC)
