# Supplementary material for: Risk Perception and Its Influence on Pre-Exposure Prophylaxis (PrEP) Uptake and Adherence Among Pharmacy-Based PrEP Users in South Africa: A Qualitative Study
Source: J Int Assoc Provid AIDS Care. 2026 Jun 9;25:23259582261447318. doi: 10.1177/23259582261447318 (PMC13519221; doi:10.1177/23259582261447318)
Supplement: Supplementary file 1 [file sj-pdf-1-jia-10.1177_23259582261447318.pdf]

Consolidated criteria for reporting qualitative studies (COREQ): 32-item checklist

Developed from:
Tong A, Sainsbury P, Craig J. Consolidated criteria for reporting qualitative research (COREQ): a 32-item checklist for interviews and focus groups. *International Journal for Quality in Health Care*. 2007. Volume 19, Number 6: pp. 349 – 357

MANUSCRIPT TITLE:

**No. Item**	**Guide questions/description**	**Reported on Page #**
**Domain 1: Research team and reflexivity**		
Personal Characteristics		
1. Inter viewer/facilitator	Which author/s conducted the interview or focus group?	Title page
2. Credentials	What were the researcher's credentials? (E.g. PhD, MD)	Title page
3. Occupation	What was their occupation at the time of the study?	Title page
4. Gender	Was the researcher male or female?	Title page
5. Experience and training	What experience or training did the researcher have?	Title page
Relationship with participants		
6. Relationship established	*Was a relationship established prior to study commencement?* .	5/6
7. Participant knowledge of the interviewer	*What did the participants know about the researcher? (e.g. personal goals, reasons for doing the research).*	5/6
8. Interviewer characteristics	*What characteristics were reported about the interviewer/facilitator? (e.g. Bias, assumptions, reasons and interests in the research topic)*	5/6
**Domain 2: Study design**		
Theoretical framework		
9. Methodological orientation and Theory	*What methodological orientation was stated to underpin the study? (e.g. grounded theory, discourse analysis, ethnography, phenomenology, content analysis).*	3/4

Information Classification: General

Participant selection		
10. Sampling	*How were participants selected? (e.g. purposive, convenience, consecutive, snowball)*	5/6
11. Method of approach	*How were participants approached? (e.g. face-to-face, telephone, mail, email)*	5/6
12. Sample size	*How many participants were in the study?*	8
13. Non-participation	*How many people refused to participate or dropped out? Reasons?*	6
Setting		
14. Setting of data collection	*Where was the data collected? (e.g. home, clinic, workplace)*	5
15. Presence of non-participants	*Was anyone else present besides the participants and researchers?*	5
16. Description of sample	*What are the important characteristics of the sample? (e.g. demographic data, date)*	9
Data collection		
17. Interview guide	*Were questions, prompts, guides provided by the authors? Was it pilot tested?*	7
18. Repeat interviews	*Were repeat interviews carried out? If yes, how many?*	5
19. Audio/visual recording	*Did the research use audio or visual recording to collect the data?*	7
20. Field notes	*Were field notes made during and/or after the interview or focus group?*	7
21. Duration	*What was the duration of the inter views or focus group?*	7
22. Data saturation	Was data saturation discussed?	8
23. Transcripts returned	*Were transcripts returned to participants for comment and/or correction?*	-
**Domain 3: analysis and findings**		
Data analysis		
24. Number of data coders	How many data coders coded the data?	7

Information Classification: General

25. Description of the coding tree	*Did authors provide a description of the coding tree?*	9/10/11
26. Derivation of themes	*Were themes identified in advance or derived from the data?*	7
27. Software	*What software, if applicable, was used to manage the data?*	7
28. Participant checking	*Did participants provide feedback on the findings?*	-
Reporting		
29. Quotations presented	*Were participant quotations presented to illustrate the themes/findings? Was each quotation identified? (e.g. participant number)*	Results
30. Data and findings consistent	*Was there consistency between the data presented and the findings?*	Results
31. Clarity of major themes	*Were major themes clearly presented in the findings?*	Results
32. Clarity of minor themes	Is there a description of diverse cases or discussion of minor themes?	Results

Information Classification: General
