# Supplementary material for: Risk Perception and Its Influence on Pre-Exposure Prophylaxis (PrEP) Uptake and Adherence Among Pharmacy-Based PrEP Users in South Africa: A Qualitative Study
Source: J Int Assoc Provid AIDS Care. 2026 Jun 9;25:23259582261447318. doi: 10.1177/23259582261447318 (PMC13519221; doi:10.1177/23259582261447318)
Supplement: Supplementary file 2 [file sj-pdf-2-jia-10.1177_23259582261447318.pdf]

# Participant In-depth Interviews

Record ID ______________________________

PPREPP-SA

Version 1.2; 16 February 2023 - Initial Design

INTRODUCTION

Hello, my name is ______ ______
QUESTIONS
1. What do you think about our proposed intervention and what makes you feel this way? First reactions.

Probe for:
• PrEP use history
• Acceptability ______

2. What do you think are the key factors that will make an initiative like this a success?

Probe for:
• Staff attitudes
• Time
• Information
• Recruitment and follow-up
• Frequency of visits
• Privacy in the pharmacy where they are accessing PrEP

______

3. What challenges do you anticipate from this intervention?

Probe for:
• Privacy/confidentiality
• Time
• Cost
• Space
• Information
• Staff attitudes
• Frequency of visits
• Availability of drug

______

4. What do you think pharmacy staff need to do make the people want to start taking PrEP and to get them at the pharmacy?

Probe for:
• Staff attitudes
• Staff time
• Staff knowledge

______

5. What other strategies do you think we need to consider to make this initiative in pharmacies successful?

Probe for:
• Optics
• Friendly services
• Leaflets and their design (words, pictures, colour, language, size)
• Social media, marketing, information sharing
• Placement of information and usefulness of information

---

I'm interested in finding out what you think will make people more interested in PrEP
6. What strategies will lead to successful recruitment of participants?

Probe for:
• Information needed to give people accessing services in the pharmacy/store
• Recruitment areas (geographically and within the pharmacy/store)
• Products associated with sexual and reproductive health e.g. condoms, lubricants, pregnancy tests, HIV test
• Recruiters/ promoters

---

7. What do you think are things that people will consider when they are making the decision about starting PrEP in pharmacies?

Probe for:
• Time of procedures (including consenting)
• Investigations associated
• Cost
• Confidentiality

---

I want to ask further questions on online PrEP initiation
8. What strategies will make this is a success?

Probe for:
• Privacy
• Convenience

---

9. What challenges do you anticipate?

Probe for:
• Barriers such as cost of data, access to smartphone/laptop
• Healthcare support
• Delivery options (home, work, pick up lockers)

---

10. What factors should be considered in designing this strategy?

Probe for:
• Enablers: information, support

---

I am also interested in knowing what would encourage young women and men in particular who are at risk for getting HIV to initiate on PrEP.
11. How would you motivate young women and men to take up PrEP?

Probe for:
• Incentives or rewards
• Format (loyalty vouchers, gifts)
• Combining PrEP with other sexual and reproductive health products e.g. condoms, lubricants, pregnancy tests, HIV test, sanitary pads

---

12. Would you be willing to pay for PrEP? Do you think other people will be willing to pay for PrEP?

Probe for:
• How much
• Medical aid cover

______

## Conclusion

Thank you for taking the time to speak with me! Do you have any questions? Is there anything we did not cover in our discussion today that you would like to mention at this time? ______

## Form Completion:

Name: ______

Date first saved: ______
