# Supplementary material for: Risk Perception and Its Influence on Pre-Exposure Prophylaxis (PrEP) Uptake and Adherence Among Pharmacy-Based PrEP Users in South Africa: A Qualitative Study
Source: J Int Assoc Provid AIDS Care. 2026 Jun 9;25:23259582261447318. doi: 10.1177/23259582261447318 (PMC13519221; doi:10.1177/23259582261447318)
Supplement: Supplementary file 3 [file sj-pdf-3-jia-10.1177_23259582261447318.pdf]

In-pharmacy initiated IDI

Record ID

____

PPREPP-SA

Version 1.0; 25 January 2024 - Initial Design

Introduction to the In-depth interviews

Hello, my name is ______ and I am a researcher at Ezintsha, an organisation associated with the University of the Witwatersrand. Thank you for agreeing to take part in this interview. This interview will help us evaluate the effectiveness and feasibility of the pharmacy-led and online-based pre-exposure prophylaxis (PrEP) initiation programme. Your participation in this interview will enable us to target areas that need improvement and any findings from this research could result in improvement of PrEP implementation that directly benefits prevention strategies in South Africa.

This interview should take approximately 1 hour. I will take written notes of our discussion and would like to audio record the interview, with your permission, to be sure that I have accurately and completely captured your responses. The notes and recordings of our discussion will be transcribed and analysed only by other members of our research team after your name and other identifying information are removed from the transcripts. Your interview information will remain confidential and secure throughout the entire programme. The Health Professions Council of South Africa (HPCSA) regulations restrict audio recordings to be kept for two years after publication and six years for no publication.

Your participation is completely voluntary. If you participate you may choose not to answer some of the questions or to discontinue the interview at any time for any reason.

[Start recording for telephonic interviews]

[In person interviews only: Go over and have them sign the consent form. Remember to provide them with a copy.]

Do you have any questions before we begin the interview?

[Answer any questions they may have and then begin.]

Pharmacy client/participant group categories

*** Participants/Pharmacy clients who initiated PrEP and are still active
*** Participants/Pharmacy clients who initiated and dropped out of the study.
*** Participants/Pharmacy clients who declined PrEP initiation
[Demographics to be completed on REDCap prior to interview recording]

Interviewer to ask - Do you consent to being recorded as indicated during the informed consent process?

Response expected - Yes.

[Start recording for in person interviews]

Section A - Health seeking behaviour

For all categories of participants:

1. Which pharmacies do you normally access services from?

Probe: Retail, privately owned, government pharmacies (Clicks, Dis-Chem, Spar, local/community pharmacies, public or private facilities)

2. What services did you access through pharmacies prior to the PrEP programme?

Probe: Health and pharmacy (self medication, prescription, vaccination, wellness checks, vitamins, contraceptives, STIs) toiletries, luxury beauty, beauty care, accompanying someone, no use of pharmacies before programme).

3. What are your sexual health seeking behaviour preferences or tendencies (contraceptives, condoms, PEP, morning after pill, PrEP, none), and where are you seeking services?
Probe: Location, only if the answer to question 2 is "no use of pharmacies before programme".

4. How can you describe your sexual behaviour (linked to risk perception)
a. Number of sexual partners, prevention preferences, HIV status of partners, types of sex (vaginal/anal), substance abuse and sex? (With whom is prevention used?)
b. Do you make it a habit to test for HIV with your sexual partner(s) and, why, why not? If yes, please tell us your partner(s) current HIV status.
c. Do you consider yourself a suitable candidate for PrEP? Why, why not? (PrEP knowledge, and where information was received).

5. Have you ever had an STI, if yes, do you know how you got it?
a. How did you handle/deal with the STI? (Name of STI or symptoms)
b. When was, if at all, the most recent time you thought you were at risk of HIV and/STIs?

Section B - Views about the programme

6. In general, what do you think about offering PrEP in pharmacies? What makes you feel this way? (What they think of it as a service/offering).

Probe: Convenience.

Section C - Motivation to participate in the programme.

For Categories 1 and 2 ONLY: (Initiated)

7. Please share what made you decide to participate in the programme?

8. What has been your experience participating in this programme?

a. Experience with initiating PrEP (testing process, counselling, anything else)
b. Availability of time for scheduled visits
c. Frequency of follow up visits.
d. Cost implications for accessing PrEP - e.g., Transport
e. Healthcare worker interactions
f. Support between refills

9. What has been your experience of using PrEP, from initiating till present?

a. Side effects
b. Adherence (pill size, frequency of refills, frequency of use, ease of use, packaging)
c. Disclosure

10. Please talk to us about your views on the pharmacy's ability to provide PrEP.

a) Staff attitudes
b) Perception of staff training
c) Pharmacy infrastructure (space)
d) Drug stocking/availability
e) Privacy in the pharmacy where they are accessing PrEP.
f) Staff availability/ waiting times.
g) Time it takes/took to initiate (clinical process such as testing etc)

h) Screening tests - HIV, pregnancy etc (comfort with screening tests, the type of tests conducted, thoughts on the screening test process)

For Category 2 ONLY - (dropped out)

11. Can you please share what made you drop out of the programme? (Sexual activity, risk perception, pill burden, programme related issues like waiting times, testing process, staff attitude, time availability, any other)

12. How do you think you can be motivated and encouraged to continue to take PrEP (i.e., retention)? Probe for programme related factors like incentives.

For Category 3 ONLY - (declined)

13. Please tell us, what made you decide not to participate in the programme? (May indicate lack of sexual activity. Probe perceived risk)

14. How do you think you can/could've been motivated and encouraged to take PrEP? (Probe on role of incentives)

Section D - PrEP knowledge and preference

For all categories of participants

15. What is your opinion about the communication and information provided by the study team?

Probe: Did the information and communication provided influence your decision for study participation?
a. What new information about PrEP did you acquire through this programme?
b. How would you say this programme has impacted on your knowledge about PrEP?

16. There are different ways of taking contraceptives such as IUD, pills, injection. Do you know if there are other options for taking PrEP? If yes, what are they?
If they do not know, moderator must provide the options (Oral PrEP, Injectable PrEP, Ring)

17. Knowing that you have options, which do you think would be your preferred PrEP option? Why?

Section E - Participants overall recommendations

For all categories of participants

18. What do you think can be done to improve the delivery of PrEP through pharmacies?
a. How to increase enrolment and ensure adherence
b. Strategies that can be implemented for pharmacies to sustain PrEP delivery.

19. Have you shared information about this programme and/PrEP with anyone? If yes, how did you share this information?

20. Would you recommend the programme and/PrEP to anyone? (Probe for reasons).

Conclusion

Thank you for taking the time to speak with me! Do you have any questions? Is there anything we did not cover in our discussion today that you would like to mention at this time?

Name: ______________________________

Date first saved: ______________________________
